# Safety and efficacy of Holmium laser enucleation of the prostate (HoLEP) in patients with previous transperineal biopsy (TPB): outcomes from a dual-centre case-control study

**DOI:** 10.1186/s12894-019-0523-z

**Published:** 2019-10-22

**Authors:** Chris Bell, Sacha L. Moore, Amarit Gill, Obinna Obi-Njoku, Stephen F. Hughes, Asad Saleemi, Gidon Ellis, Farooq Khan, Iqbal S. Shergill

**Affiliations:** 1 Department of Urology, Wrexham Maelor Hospital, Wrexham, Wales, UK; 2 North Wales Clinical Research Centre, Gwenfro, Wrexham Technology Park, Wrexham, LL13 7YP UK; 3 Department of Urology, Luton and Dunstable University Hospital, Lewsey Road, Luton, LU4 0DZ UK

**Keywords:** Holmium laser Enucleation of the prostate (HoLEP), Benign prostatic enlargement (BPE), Transperineal template biopsy, Prostate, Lower urinary tract symptoms (LUTS), Urinary retention

## Abstract

**Background:**

We investigated the surgical feasibility, safety and effectiveness of 50 W (low power) Holmium Laser enucleation of the prostate (HoLEP) in patients who have undergone previous template biopsy of the prostate (TPB).

**Methods:**

Data encompassing pre-operative baseline characteristics, intra-operative measures and post-operative outcomes was collected for 109 patients undergoing HoLEP across two UK centres. Patients were stratified into two groups; group 1 (*n* = 24) had undergone previous TPB were compared with ‘controls’ (no previous TPB) in group 2 (*n* = 85). The primary outcome was successful HoLEP.

**Results:**

There were no statistically significant differences in either key baseline characteristics or mass of prostate enucleated between groups 1 and 2. There was no statistically significant difference in enucleation or morcellation times parameters between the two groups other than enucleation efficiency in favour of group 1 (*p* = 0.024). Functional outcomes improved, without any statistically significant difference, in both groups.

**Conclusions:**

In patients with a previous TPB, HoLEP is surgically feasible, safe and effective. TPB should not be considered a contraindication to HoLEP. Our work provides a strong foundation for further research in this area.

## Background

The advent of Holmium laser enucleation of the prostate (HoLEP) has triggered a paradigm shift in the surgical management of benign prostatic enlargement (BPE); with improved functional outcomes, lower complication rates and length of stay, and fewer repeat procedures required at 5–10 years versus transurethral resection of the prostate (TURP), open prostatectomy and other laser therapies. HoLEP is now regarded as the gold standard for BPE [1–3]. Evidence that HoLEP is safe and efficacious in a wider patient demographic with varying co-morbidities is necessary as its use spreads.

The use of transperineal prostate biopsy (TPB) is increasing as a result of improved accuracy in diagnosing (or excluding) clinically significant cancer coupled with negligible risk of sepsis [4, 5]. However, patients undergoing TPB may rarely suffer from refractory retention post-biopsy requiring surgical management, and in addition, there is a cohort of patients who develop unrelated retention or lower urinary tract sy (LUTS) secondary to BPE after past TPB and require surgical intervention [6].

Trans-rectal ultrasound (TRUS) guided biopsies have been performed prior to HoLEP and have not been shown to affect the technical difficulty of the procedure [7–10]. However TPB has important differences including more extensive sampling of the transitional zone versus TRUS-guided biopsies. Due to this theoretical disruption of prostate architecture during TPB many surgeons feel that HoLEP may be more challenging in this cohort of patients and that operative and functional outcomes could be compromised.

Whilst recent literature has demonstrated HoLEP’s use following previous prostate surgery such as TURP and prostatic urethral lift surgery without compromise, and in those undergoing active surveillance for prostate cancer or with very large prostates, to our knowledge no published data evaluating outcomes of HoLEP after TPB exists [11–13]. As such in this study, we aimed to assess the feasibility, safety, and efficacy of HoLEP in patients who had undergone previous TPB.

## Methods

### Data collection

Following local institution audit registration (reg no. 18/171) we conducted a dual centre analysis of men undergoing 50 W low-power HoLEP between January 2016 and October 2018. Patients were stratified into two groups; group 1 had undergone TPB prior to HoLEP, while group 2 men were biopsy-naïve (“controls”). Patients in group 1 were from two centres whereas “control” patients in group 2 were from the second centre only. This was due to the fact that group 2 patients were consecutive patients from early in the learning curve from one surgeon – hence this would serve as the baseline control. Both groups had several surgeons performing the procedure. Patients who had undergone any prostate surgery prior to HoLEP or TPB were excluded. Data was collected into an Excel database, with both electronic and paper notes retrospectively reviewed to address missing data where possible and to gather information on post-operative outcomes.

Collected data was categorised according to timepoint relative to HoLEP. Pre-HoLEP measures included indication for HoLEP, time from TPB to HoLEP, results of TPB, prostate specific antigen (PSA) level, prostate volume measured using trans-rectal ultrasound (TRUS), haemoglobin, and creatinine. International prostate symptoms scores (IPSS) including quality of life score (QoL) and uroflowmetry were recorded where possible and relevant. Intra-operative measures included lobes enucleated, mass enucleated, enucleation time, morcellation time, morcellation efficiency, and whether there was a conversion to TURP. Post-HoLEP measures included length of hospital stay, post-operative complications (indexed as per the Clavien-Dindo classification), and histological findings of enucleated tissue.

### Procedure information

Template biopsies were taken as per the PROMIS trial method [14].All included patients underwent low-power HoLEP using 50 W Auriga XL laser (Boston Scientific Inc.), under general anaesthesia, according to previously well-described HoLEP technique [15]. Morcellation was performed with the Wolf Piranha Morcellator system, and the use of bipolar diathermy for haemostasis was performed according to the discretion of the operating surgeon.

### Data analysis

The primary outcome measure was successful HoLEP. Success was defined according to outcome at follow-up depending on indication. For those undergoing HoLEP for LUTS, success was defined by improvement in post-operative IPSS of ≥6, or improvement in quality of life (QoL) score of ≥2 points. A similar improvement in IPSS has been considered successful in other studies [16]. In patients without a pre-operative IPSS+QoL for comparison, success was defined as a post-operative QoL of ≤2 at follow-up. In patients with LUTS but no documented IPSS pre- or post-operatively, success was defined as no need for further medication or intervention for LUTS by 6 months. In patients undergoing HoLEP for refractory retention, success was defined as ability to void spontaneously.

Data was analysed using SPSS version 25 for Windows (IBM Corp., Armonk, NY). Where data was normally distributed, parametric analysis was undertaken employing the student’s T-test, while Mann-Whitney U testing was performed for non-parametric continuous data. Differences between categorical variables were calculated using chi-square tests. Confidence intervals were calculated using the Newcombe-Wilson method without continuity correction. Outcomes were reported in accordance with the STROBE statement [17].

## Results

### Demographic and baseline data

Over the 34-month period, 24 men were identified who had undergone TPB prior to HoLEP (group 1), and 85 men were identified in group 2. Table 1 demonstrates baseline demographic and pre-operative characteristics. The mean age of patients was 66.8 years in group 1 and 71.8 years in group 2 95% CI 1.2–8.9, *p* = 0.012). The median time from TPB to HoLEP was 38.6 weeks (range 7–163 weeks, IQR 17.6–79.4). In Group 1, 13 patients had undergone HoLEP for refractory urinary retention versus 51 in group 2 (not statistically significant), while 11 patients in group 1 underwent HoLEP for LUTS, as compared to 34 patients in Group 2 (not statistically significant). Mean length of hospital stay was 1.6 days (range 1–8), and differed significantly between the two groups (1.3 days for group 1 versus 3.0 days for group 2, *p* < 0.001). Information on pre-operative medications is presented in Additional file 1: Table S1.

**Table 1:** pre-operative baseline data

	Group 1	Group 2	Difference (95% CI)	*p* value
Age (years)	66.8 (±8.2)	71.8 (±8.7)	5.0 (1.2, 8.9)	0.012
TRUS volume (cm^3^)	76.1 (±35.0)	69.3 (±31.8)	8.5 (−24.1, 10.6)	0.402
PSA (ng/mL)	10.2 (±5.7)	5.0 (±3.9)	5.1 (2.5, 7.8)	< 0.001
Indication for HoLEP				
LUTS	11 (45.8%)	34 (40.0%)	5.8 (−15.0, 27.3)	0.644
Refractory retention	13 (54.2%)	51 (60.0%)		
*Q*_max_ (mL/sec)	9.9 (±4.3)	9.6 (±5.2)	0.4 (−3.0, 3.8)	0.817
PVR (mL)	181.4 (±99.6)	176.8 (±140.3)	4.6 (− 77.5, 86.8)	0.908
IPSS	22.4 (±4.7)	22.6 (±6.9)	0.2 (−3.7, 4.0)	0.923
Quality of life score	4.1 (±1.0)	4.4 (±1.2)	0.3 (−0.5, 1.1)	0.484
Serum creatinine (μmol/L)	81.0 (±16.0)	86.7 (±30.3)	5.7 (−3.5, 15.0)	0.219

*CI* Confidence intervals, *TRUS* Trans-rectal ultrasound, *PSA* Prostate specific antigen, *HoLEP* Holmium laser enucleation of the prostate, *LUTS* Lower urinary tract symptoms, *Q*_*max*_ maximum flow rate, *PVR* Post-void residual volume, *IPSS* International Prostate Symptom Score. Data presented as mean (SD) except *counts (%).

Of the 24 men in group 1 who underwent TPB, the mean number of biopsies per patient was 40.5 (IQR 32.5–58.75). 16/24 (67%) of patients had benign histology whilst 8/24 (33%) of patients had Gleason score ≥ 3 + 3 (3 + 3, *n* = 7; 3 + 4, *n* = 1), of which five were unilateral and three bilateral. Additional information on the template biopsy histology is presented in Additional file 2: Table S2.

Pre-operative IPSS scores were available for 11/24 (45.8%) patients in group 1 and 33/85 (38.8%) patients in group 2. Pre-operative maximum flow rate (*Q*_*max*_) and post-void residual (PVR) was available for 10/24 (41.7%) patients in group 1 and 35/85 (41.2%) patients in group 2. There were no significant differences at baseline between the two groups in key pre-operative parameters including mean prostate volume, *Q*_*max*_, PVR, and IPSS score. Baseline PSA was significantly different between the two groups (10.2 versus 5.7, *p* < 0.001).

### Operative outcomes in biopsied versus biopsy-naive men

Table 2 displays intra-operative outcomes. There was no significant difference in the mass enucleated (mean 54.7 g in group 1 vs 44.2 g in group 2, 95% CI -4.8, 25.7, *p* = 0.172). Notably, enucleation and morcellation time were comparable between the two groups; enucleation time was 55.8 min in group 1 versus 59.7 min in group 2 (95% CI -7.6, 15.5, *p* = 0.172), while morcellation time was 12.7 min in group 2 versus 10.1 min in group 2 (95% CI -1.9, 7.2, *p* = 0.248). Enucleation efficiency was significantly improved in group 1 versus group 2 (1.05 g/min versus 0.76 g/min, 95% CI 0.04,0.54, *p* = 0.024), however there was no significant difference in morcellation efficiency (5.04 g/min versus 6.59 g/min, 95% CI -0.30, 3.39, *p* = 0.100). Use of bipolar diathermy was also comparable and minimal, and there were no conversions to TURP. Three surgeons operated on a similar number of patients each (*n* = 43, 34, 32 respectively), with no difference in enucleation efficiency (*p* = 0.541) or morcellation efficiency (*p* = 0.581) between surgeons.

**Table 2:** intra-operative measures

	Group 1	Group 2	Difference (95% CI)	*p* value
Mass enucleated (g)	54.7 (±33.1)	44.2 (±29.6)	10.5 (− 4.8, 25.7)	0.172
Enucleation time (mins)	55.8 (±24.0)	59.7 (±24.4)	3.9 (−7.6, 15.5)	0.496
Enucleation efficiency (g/min)	1.05 (±0.50)	0.76 (±0.61)	0.29 (0.04, 0.54)	0.024
Morcellation time (mins)	12.7 (±9.5)	10.1 (±7.8)	2.6 (−1.9, 7.2)	0.248
Morcellation Efficiency (g/min)	5.04 (±1.45)	6.59 (±7.69)	1.55 (−0.30, 3.39)	0.100

*CI* Confidence intervals. Data presented as mean (SD) except *counts (%).

Post-operative outcomes for both cohorts are described in Table 3. All HoLEPs in group 1 were deemed successful, while 91% of HoLEPs in group 2 were successful.

**Table 3:** post-operative measures

	Group 1	Group 2	Difference (95% CI)	*p* value
*Q*_max_ (mL/sec)	17.3 (±6.7)	22.2 (±15.1)	5.1 (− 4.1, 14.1)	0.264
PVR (mL)	70.9 (±121.6)	63.1 (±84.4)	7.8 (−76.1, 91.7)	0.847
IPSS	6.2 (±4.5)	6.2 (±5.8)	0.1 (−2.9, 2.9)	0.990
Quality of life score	1.0 (±0.8)	1.5 (±1.74)	0.5 (−0.1, 1.1)	0.116
Mean hospital stay (days)	1.3 (1.0)	3.0 (3.6)	1.7 (0.45, 0.77)	< 0.001

*CI* Confidence intervals, *Q*_*max*_ maximum flow rate, *PVR* Post-void residual volume, *IPSS* International Prostate Symptom Score. Data presented as mean (SD).

Post-operative IPSS and QoL scores were available for 14/24 (58.3%) patients in group 1 and 61/85 (71.8%) patients in group 2. Post-operative *Q*_*max*_ and PVR was available for 12/24 (50.0%) patients in group 1, whilst *Q*_*max*_ was available for 15/85 (17.6%) patients and PVR was available for 20/85 (23.5%) patients in group 2. There were no significant differences between groups 1 and 2 in post-operative *Q*_*max*_ (17.3 mL/sec vs 22.2 mL/sec, 95% CI -4.1, 14.1, *p* = 0.264), PVR (70.9 mL versus 63.1 mL, 95% CI-76.1, 91.7, *p* = 0.847), IPSS (6.2 versus 6.2, 95% CI -2.9, 2.9, *p* = 0.990) or QoL score (1.0 versus 1.5, 95% CI -0.1, 1.1, *p* = 0.116). Additionally, pre-HoLEP TPB result was compared with each of these outcome measures. Although numbers are too small to be conclusive, there was no significant difference in post-operative *Q*_*max*_, PVR, IPSS or QoL score in the eight patients with a previous positive biopsy compared with an age-matched representative sample of the cohort. Time to HoLEP had no effect on enucleation and morcellation efficiency on correlation analysis (*p* = 0.326 & 0.604 respectively).

24/109 (22.0%) patients were documented as having a complication post-HoLEP (4/24 in group 1 and 20/85 in group 2). All complications were Clavien-Dindo 1 or 2. 14/24 (58.3%) patients had temporary stress urinary incontinence as their sole complication. Seven patients (6.4% of total cohort) reported erectile dysfunction post-operatively in clinic. One patient required a blood transfusion. Four patients required a catheter on discharge, but these were all successfully removed with spontaneous voiding at 14, 22, 30, and 45 days. No patients required medications post-operatively.

All patients in both cohorts had samples sent from HoLEP for histology. 4/24 (16.7%) patients in group 1 had positive histology; seven patients had Gleason 3 + 3 disease with one patient having Gleason 3 + 4. 10/85 (11.8%) patients in group 2 had positive histology; eight patients had Gleason 3 + 3 disease, one patient had Gleason 3 + 4, and one patient had Gleason 5 + 4. One patient with Gleason 3 + 3 disease and the patient with Gleason 5 + 4 disease had a pre-existing diagnosis of prostate cancer and were on hormone therapy. There was no statistically significant difference between incidental detection of prostate cancer between the two groups (*p* = 0.402). Additional information on histological features in presented in Additional file 3: Table S3. Histology post-HoLEP was reviewed alongside complications; there was no significant difference in complication rate between benign and malignant groups. Additionally, pre-HoLEP TPB histology in group 1 was shown not to be significantly associated with complication rate.

### Functional outcomes in biopsied versus biopsy-naive men

**Fig. 1:**
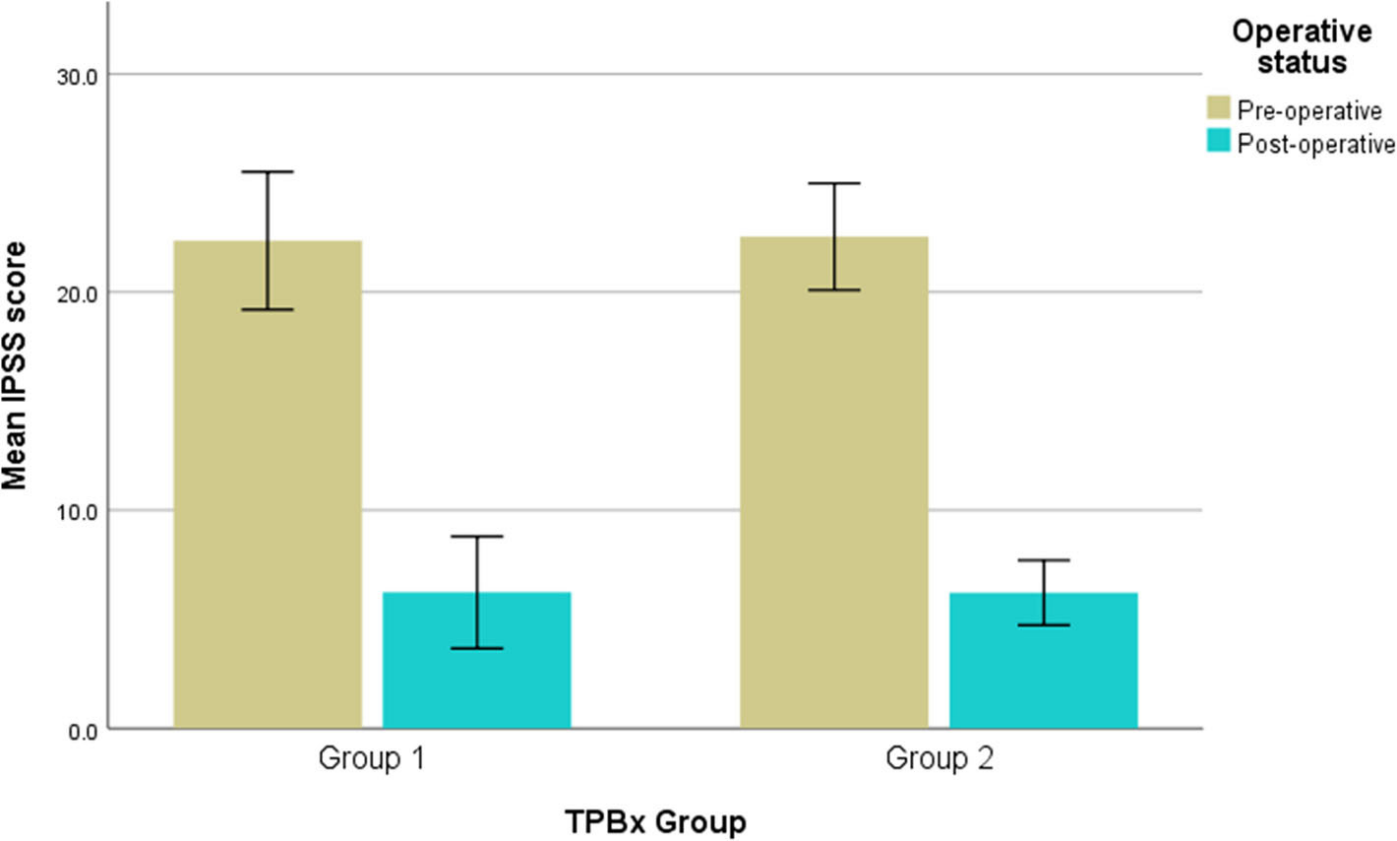
Comparison of mean IPSS scores pre and post-HoLEP between those with previous template biopsy (group 1) and those without previous prostate intervention (group 2). Error bars denote 95% confidence intervals. IPSS: International Prostate Symptom Score; TPB: transperineal template biopsies

**Fig. 2:**
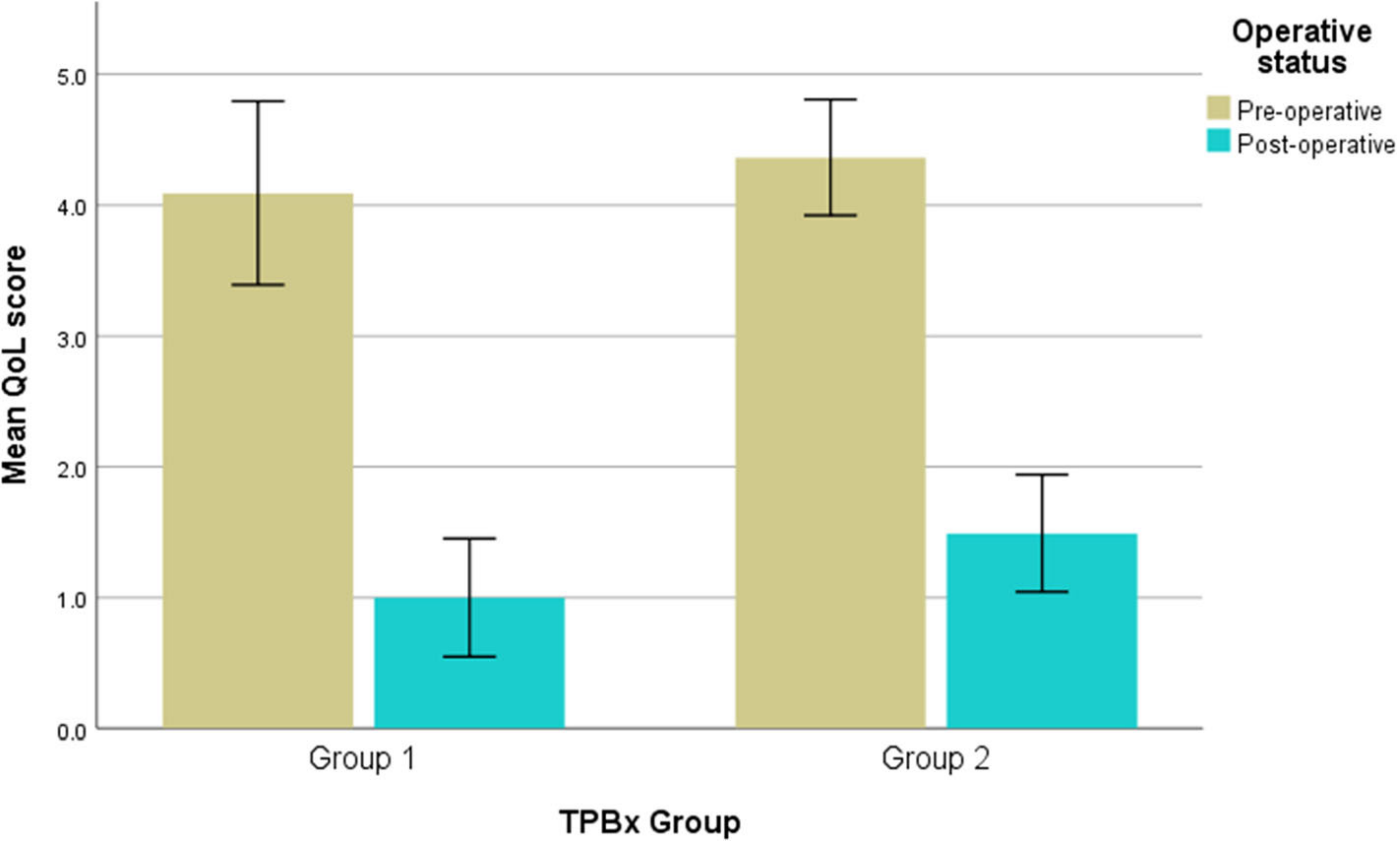
Comparison of mean quality of life scores pre and post-HoLEP between those with previous template biopsy (group 1) and those without previous prostate intervention (group 2). QoL: quality of life score

Pre- and post-operative IPSS were compared in both groups 1 and 2 (Fig. 1); mean IPSS significantly improved post-HoLEP in both groups (group 1 mean IPSS pre-operatively 22.4 vs 6.2 post-operatively, *p <* 0.001; group 2 mean IPSS pre-operatively 22.5 vs 6.2 post-operatively, *p <* 0.001). Pre- and post-operative QoL scores were also compared in both groups 1 and 2 (Fig. 2); mean QoL significantly improved post-HoLEP in both groups (group 1 mean QoL score pre-operatively 4.1 vs 1.0 post-operatively, *p <* 0.001; group 2 mean QoL score pre-operatively 4.4 vs 1.5 post-operatively, *p <* 0.001).

Pre- and post-operative *Q*_*max*_ was compared in both groups 1 and 2; mean *Q*_*max*_ significantly improved post-HoLEP in both groups (group 1 mean *Q*_*max*_ pre-operatively 9.9 mL/s vs 17.5 mL/s post-operatively, *p* = 0.007); group 2 mean *Q*_*max*_ pre-operatively 9.6 mL/s versus 22.1 mL/s post-operatively, *p <* 0.001). Pre- and post-operative PVR was also compared in both groups 1 and 2; mean PVR significantly improved post-HoLEP in both groups (group 1 mean PVR pre-operatively 181.4 mL vs 70.9 post-operatively, *p* = 0.03; group 2 mean PVR pre-operatively 176.8 mL vs 63.1 mL post-operatively, *p* = 0.002).

## Discussion

### Meaning of the study

As such, our study is the first to demonstrate that HoLEP is a safe and feasible option, following previous TPB. With increasing number of men undergoing TPB, the population we have studied represents a common cohort of prostate patients, and as such, is a vital addition to the HoLEP evidence base.

Crucially, there is a view by some that HoLEP could be more difficult in patients post-TPB due to altered architecture of prostatic tissue. Our study provides evidence to the contrary and confirms the safety and efficacy profile of HoLEP, to include men who have undergone TPB. Both groups in our study had comparable baseline characteristics, with no significant differences between key indicators including indication for HoLEP, prostate volume on TRUS, pre-operative *Q*_*max*_, PVR, IPSS and QoL score (*p* > 0.05). PSA was significantly higher in group 1 (*p* = < 0.001) but this was expected given patients in group 1 likely underwent TPB for investigation of raised PSA originally.

As is shown in Table 2, there was no significant difference in key intra-operative markers. If, as hypothesized, TPB led to an altered texture of prostate tissue and hence a more challenging HoLEP surgical procedure, it would be expected that in these cases enucleation and morcellation would take longer, which was not the case. Enucleation efficiency was, in fact, higher (*p* = 0.024) in the previous TPB group; we cannot provide evidence as to why this might be as to date there have not been any published studies in this area, however it is possible that there are physiological changes to prostate tissue post-biopsy and thus this is an area that warrants further research. The data therefore supports our assertion that is not made more technically challenging by a previous TPB.

Table 3 highlights no significant difference in key post-operative outcomes between the two groups, with marked improved pre- versus post-HoLEP in both groups. Additionally, all procedures in group 1 were deemed successful with a statistically significantly shorter length of hospital stay. Functionally, it is clear that HoLEP significantly improved both patient reported outcomes and objective outcomes regardless of previous TPB status, and thus is clearly an effective treatment option in both of these patient groups.

Although complication rates were slightly higher than reported in the literature [18], it is worth noting that the majority of these were temporary stress urinary incontinence which resolved. It is also worth noting that patients in group 2 (where complication rate was higher compared to group 1) were all from one centre where the operating surgeon had just completed their learning curve [19]. Thus, this allowed for comparison between the potential challenge of patients post-biopsy in group 1 against control patients operated on by a surgeon with less experience in HoLEP.

### Strengths and limitations

To our knowledge, this study provides the first evidence in the literature of the feasibility of HoLEP in those who have undergone TPB. The strength of our study lies in its provision of a breadth of data over two centres in two groups with comparable baseline characteristics. There were no significant differences between the groups recorded in key demographic and preoperative fields that might confound the results.

Nevertheless, we acknowledge that there are a number of limitations to our study. Although the data was largely collected from a prospectively-maintained database, some of the data (mainly post-operative measures) were retrospectively gathered and thus this opens up the possibility of misclassification bias. Likewise, data on IPSS/QoL scores in some patients with LUTS was missing either partially (i.e. only a pre-operative or post-operative score) or in its entirety. Consequently we were unable to quantify the degree of improvement in symptoms via this validated tool in a subgroup of patients. However, in this subgroup we used a pragmatic surrogate measure of success (no need for further medication or intervention for LUTS by 6 months) which we feel confers at least an acceptable level of accuracy.

Our study also focused on short-term post-operative outcome data and thus our study may miss long-term need for re-operation or longer term complications. There is also a large range of time from TPB to HoLEP, which could have an effect on patient expectations and therefore, potentially, outcomes.

Additionally, our study used 50 W low-power HoLEP, so application of these results to higher-power procedures has to be used with some degree of caution. The possible altered experience and adverse effect profile with higher-power lasers may mean differences in previously biopsied prostates become more prominent.

### Recommendations for future research

Given this is the first study of its kind in this cohort of patients, there would be merit in building on this foundation further with fully-prospective multi-centre studies conducting an analysis of their patients in a similar way. Additionally, studies assessing long-term outcomes in patients that have undergone HoLEP post-TPB would be of value.

## Conclusion

Our study demonstrates that 50 W low-power HoLEP is a feasible, safe and effective treatment modality for benign prostatic enlargement in patients with previous transperineal biopsy. Hence, transperineal prostate biopsy should not be considered a contraindication to HoLEP in men with benign prostatic enlargement. Further research building on this novel foundation is required to evaluate longer-term outcomes.

## Supplementary information

**Additional file 1:** **Table S1.** Preoperative medications in patients undergoing HoLEP for lower urinary tract symptoms.

**Additional file 2:** **Table S2.** Histological detail of pre-HoLEP transperineal template biopsies of the prostate.

**Additional file 3:** **Table S3.** Histological features of post-transperineal template biopsy HoLEPs.

## Data Availability

The datasets used and/or analysed during the current study are available from the corresponding author on reasonable request.

## Abbreviations

BPE: Benign prostatic enlargement
HoLEP: Holmium laser enucleation of the prostate
IPSS: International prostate symptoms scores
LUTS: Lower urinary tract symptoms
PSA: Prostate specific antigen
PVR: Post-void residual volume
*Q*_*max*_: Maximum flow rate
QoL: Quality of life score
TPB: Transperineal prostate biopsy
TRUS: Trans-rectal ultrasound
TURP: Transurethral resection of the prostate
