# Supplementary material for: Safety and efficacy of Holmium laser enucleation of the prostate (HoLEP) in patients with previous transperineal biopsy (TPB): outcomes from a dual-centre case-control study
Source: BMC Urol. 2019 Oct 22;19:97. doi: 10.1186/s12894-019-0523-z (PMC6805368; doi:10.1186/s12894-019-0523-z)
Supplement: Supplementary file 1 — Additional file 1: Table S1. Preoperative medications in patients undergoing HoLEP for lower urinary tract symptoms. [file 12894_2019_523_MOESM1_ESM.docx]

***Supplementary table 1 – preoperative medications in patients undergoing HoLEP for lower urinary tract symptoms***

| **Preoperative medication for LUTS** | **Frequency** | **(%)** |
| --- | --- | --- |
| Alpha-blocker | 21 | (46.7) |
| 5-alpha reductase inhibitor | 2 | (4.4) |
| Anti-muscarinic | 1 | (2.2) |
| Alpha-blocker plus 5-alpha reductase inhibitor | 12 | (26.7) |
| Alpha blocker plus anti-muscarinic | 3 | (6.7) |
| No medication | 6 | (13.3) |

*HoLEP, Holmium laser enucleation of the prostate; LUTS, lower urinary tract symptoms.*
