# Supplementary material for: Safety and efficacy of Holmium laser enucleation of the prostate (HoLEP) in patients with previous transperineal biopsy (TPB): outcomes from a dual-centre case-control study
Source: BMC Urol. 2019 Oct 22;19:97. doi: 10.1186/s12894-019-0523-z (PMC6805368; doi:10.1186/s12894-019-0523-z)
Supplement: Supplementary file 2 — Additional file 2: Table S2. Histological detail of pre-HoLEP transperineal template biopsies of the prostate. [file 12894_2019_523_MOESM2_ESM.docx]

***Supplementary table 2 – histological detail of pre-HoLEP transperineal template biopsies of the prostate***

| **Unilateral / bilateral** | **Gleason score** | **Number of positive cores** | **Core involvement** |
| --- | --- | --- | --- |
| bilateral | 3+3 | 3/8 | 7% |
| unilateral | 3+3 | 1/10 | 1% |
| unilateral | 3+3 | 1/10 | 1mm |
| unilateral | 3+3 | 2/10 | 2mm |
| unilateral | 3+3 | 1/10 | 1mm |
| bilateral | 3+3 | 2/10 | 3mm |
| unilateral | 3+4 | 1/10 | 1mm |
| unilateral | 3+3 | 2/10 | 2mm |

*HoLEP, Holmium laser enucleation of the prostate. Each row represents one patient.*
