# Supplementary material for: Safety and efficacy of Holmium laser enucleation of the prostate (HoLEP) in patients with previous transperineal biopsy (TPB): outcomes from a dual-centre case-control study
Source: BMC Urol. 2019 Oct 22;19:97. doi: 10.1186/s12894-019-0523-z (PMC6805368; doi:10.1186/s12894-019-0523-z)
Supplement: Supplementary file 3 — Additional file 3: Table S3. Histological features of post-transperineal template biopsy HoLEPs. [file 12894_2019_523_MOESM3_ESM.docx]

***Supplementary table 3 – histological features of post-transperineal template biopsy HoLEPs***

| **Histological feature** | **Frequency reported** |
| --- | --- |
| stromal hyperplasia | 15 |
| chronic inflammation | 11 |
| glandular hyperplasia | 10 |
| basal cell hyperplasia | 8 |
| acute inflammation | 4 |
| glandular atrophy | 1 |
| microlithiasis | 1 |
| squamous metaplasia | 1 |

*HoLEP, Holmium laser enucleation of the prostate. Excludes features of malignancy.*
